# A Case Report of Inguinal Hernia Sac Lithiasis

**DOI:** 10.1155/carm/6658363

**Published:** 2025-03-19

**Authors:** Kiana Babaei, Ali Movahedi, Azam Gazerani, Navid Soroush

**Affiliations:** ^1^Department of Anesthesia, Neyshabur University of Medical Sciences, Neyshabur, Iran; ^2^Department of Nursing, Neyshabur University of Medical Sciences, Neyshabur, Iran; ^3^Department of General Surgery, Neyshabur University of Medical Sciences, Neyshabur, Iran

**Keywords:** hernia sac lithiasis, hernioplasty, inguinal hernia

## Abstract

We report a case of a stone in inguinal hernia sac. A 41-year-old male patient presented to Hakim Hospital in Neyshabur city, Iran, with complaints of pain and swelling in the right abdominal region. After initial evaluations, a diagnosis of right inguinal hernia was made, and the patient was scheduled for hernioplasty. He had no history of previous surgeries, hospital admissions, underlying diseases, or kidney and gallbladder stones. The surgery was performed. The hernia sac was exposed. Inside the hernia sac, a stone measuring approximately 2 cm in diameter, with a hard consistency and yellow color, was found. The stone was not adherent to the sac and was mobile. The hernia sac was opened, the stone was removed, and sent to the pathology lab. The patient was discharged 24 h later in good general condition. According to the pathology report, the components of the stone were identified as calcium oxalate.

## 1. Introduction

Inguinal hernia repair is one of the most common surgical interventions [1], with varying rates across different countries. The incidence of this surgery is reported to be 10 per 100,000 of the population in the United Kingdom and 28 per 100,000 in the United States [2, 3]. Hernias happen more frequently in certain parts of the body, such as the abdomen, groin and upper thigh area, and umbilical area [4]. While the most common contents of hernias are preperitoneal fat and abdominal organs, the search results indicate that more unusual contents, such as the bladder, Meckel's diverticulum, and a portion of the intestines' circumference (Richter's hernia), may occasionally be encountered, though these are rare occurrences [5–7]. Although the incidence of unusual contents in inguinal hernias, such as bladder stones, Meckel's diverticulum, and Richter's hernia, is generally low, specific data provide insight into their occurrence rates. Meckel's diverticulum has been reported in 0.6%–0.78% of complicated abdominal hernias. Additionally, Littre's hernia, which involves the herniation of Meckel's diverticulum, accounts for approximately 1% of cases in individuals with this congenital anomaly [8, 9]. While the precise incidence of Richter's hernia remains unclear, it is recognized as a rare but noteworthy complication in clinical practice [10]. Chronic inflammation due to persistent irritation from the hernia itself or from infections can significantly increase the risk of lithiasis. The conditions that lead to ongoing inflammation create an environment that favors mineral precipitation and stone formation. Studies have showed that chronic inflammatory processes can lead to tissue changes that promote stone development in various anatomical locations, including hernias [11]. In some cases, inguinal hernia surprises the surgical team with unexpected content [11]. Considering the abnormal conditions that surgeon may face during hernia surgery, it seems important and necessary to report abnormal and rare cases. For this reason, in this study, we report a case of a stone in the inguinal hernia sac.

## 2. Case Presentation

A 41-year-old male patient presented to Hakim Hospital in Neyshabur city in Iran, with complaints of pain and swelling in the right abdominal region. After initial evaluations, including medical history and physical examination, a diagnosis of right inguinal hernia was made, and the patient was scheduled for hernioplasty. He had no history of previous surgeries, hospital admissions, underlying diseases, or kidney and gallbladder stones. Additionally, there were no gastrointestinal or urinary symptoms, and he was not on any specific medication. Routine preoperative lab results were generally within normal ranges, except for a hematocrit (HCT) of 50.1.

**Figure 1:**
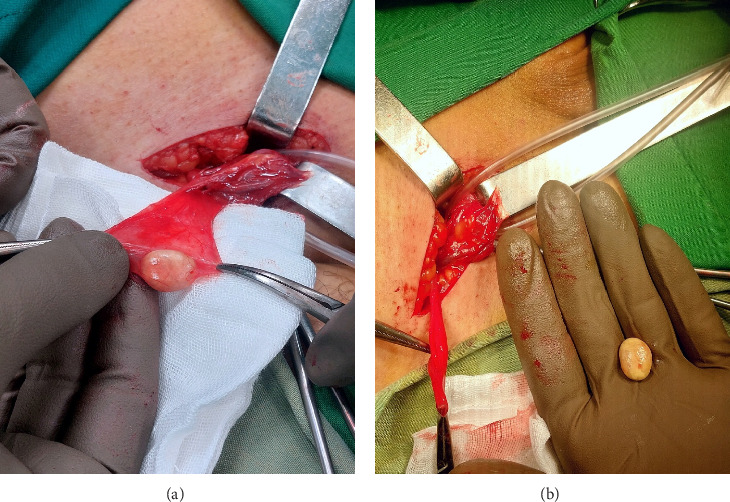
(a) Lithiasis in hernia sac and (b) stone retrieved from the hernia sac.

The surgery was performed in the supine position under spinal anesthesia. After prepping and draping (P&D) the area according to standard protocols, the procedure was commenced. An oblique incision was made in the right inguinal region. The surgical incision made in the inguinal region was approximately 9 cm in length, allowing sufficient access to the hernia sac and surrounding structures. The subcutaneous tissues and the roof of the inguinal canal were opened. The hernia sac was exposed and separated from the other components of the spermatic cord. Inside the hernia sac, a stone measuring approximately 2 cm in diameter, with a hard consistency and yellow color, was found (Figures 1(a) and 1(b)). The stone was not adherent to the sac and was mobile. The hernia sac was opened, and the stone was removed and sent to the pathology lab for chemical analysis. The cecum, appendix, and small intestine were also exposed through the hernia sac, all of which appeared normal. No free fluid was present in the abdominal cavity. The hernia sac was separated from the surrounding tissues and high ligated at the internal ring. The floor of the canal was repaired with nylon sutures, preserving the vas deferens and blood vessels, bringing the iliopubic tract closer for a triple-layer closure. The roof of the canal, skin, and subcutaneous layers were then repaired. The surgery was successfully completed, and the patient was discharged 24 h later in good general condition and stable status.

According to the pathology report, the specimen consists of a piece of cystic tissue, and the external surface of the stone was cream-colored and spherical, measuring 1.1 × 1.1 cm with a wall thickness of 0.2 cm and yellow sediments with a hard consistency. The components of the stone were identified as a fibrotic wall, which contains calcium oxalate deposits with mild vascular congestion.

Two weeks postoperatively, the sutures were removed. The patient showed no signs of infection or neuropathic pain. The overall general condition was good, with no functional limitations reported. The patient resumed daily activities without any complications.

## 3. Discussion

Inguinal hernia sacs containing stones are rare but documented occurrences. These stones can result from various causes, including spilled gallstones during laparoscopic cholecystectomy [12, 13], stone ingestion [14], or spontaneous formation within the sac [15]. Weakness in the abdominal wall is a significant predisposing factor for hernia formation, which may also create conditions favorable for stone development. This occurs due to the stasis of fluids and the increased risk of localized infection. Additionally, factors that elevate intra-abdominal pressure, such as chronic coughing or heavy lifting, can contribute to hernia formation. These pressures may also promote stone formation by causing persistent irritation and inflammation within the hernia sac. In rare cases, the presence of foreign materials within the hernia sac can act as a nidus for stone formation. Examples include surgical mesh or other materials introduced during prior medical procedures, which can provide a surface for crystallization and subsequent stone growth. Chronic inflammation associated with hernias can alter the local biochemical environment, promoting mineral precipitation and stone formation. Persistent irritation, infection, and inflammation in adjacent tissues, such as the omentum, may lead to calcification and the development of stones [15]. Inguinal hernias can present with uncommon contents, posing diagnostic and surgical challenges. Amyand's hernia, a rare variant where the appendix is found within the hernia sac, occurs in approximately 1.5% of cases [16]. This condition may be asymptomatic or lead to complications such as inflammation, incarceration, or perforation [17]. Other unusual contents may include the urinary bladder, ovary, and fallopian tubes [16].

Mazdak and Najafipour reported the first known case of an inguinal hernia containing three smooth yellowish concretions within the hernia sac. Chemical analysis revealed that these concretions contained organic material, primarily proteins, as well as calcium oxalate and calcium hydrogen phosphate crystals [11]. Similarly, Dsouza et al. presented a case involving a 56-year-old diabetic man who underwent inguinal hernia repair. During the surgery, a 2 × 2-cm stone composed primarily of calcium and oxalate was found adhered to the omentum within the hernia sac. The stone formation was likely due to chronic inflammation and calcification of the omentum within the hernia sac [15]. Another rare case presented by Sookpontaram et al. involved a 2-year-old boy who had ingested a large number of stones, leading to an obstructed inguinal hernia with perforation, which required emergency surgery. During the surgery, the surgeons found the hernia sac contained the ascending colon, which was full of the ingested stones. They had to make an additional incision to reduce the obstructed segment, which revealed a perforation, and most of the stones were removed through this perforation. Despite developing a postoperative wound infection, the boy recovered uneventfully and was discharged 10 days after the surgery [14]. Singal also contributed to the literature with a case report of a 50-year-old man with an inguinal hernia in which a stone was discovered within the hernia sac during the surgery. The authors termed this phenomenon “hernialithiasis.” The stone, measuring 1.25 cm in size, was oval-shaped and pale yellow in color. It was composed primarily of calcium (60%) and phosphate (40%), with no oxalate, urate, or cholesterol found [18].

In the present case, the patient had no history of prior surgery nor did they have gallstones or kidney stones. Considering the other reported cases, it is hypothesized that chronic retention of abdominal contents within the hernia sac, due to obstruction and reduced flow of fluids and gases, could create an environment conducive to the deposition and formation of stones. Additionally, decreased bowel motility due to the presence of the hernia could lead to increased time for mineral and sediment retention within the hernia sac, ultimately leading to stone formation. However, these hypotheses require further investigation and studies to elucidate the exact mechanisms leading to stone formation in inguinal hernia sacs. Management typically involves stone removal and hernia repair, with potential additional procedures based on complications. The reporting of such cases can assist surgeons in managing unexpected and unusual conditions, which is crucial for proper management and preventing potential complications.

## 4. Conclusion

Inguinal hernias with unusual contents such as stones are rare but clinically significant. This case highlights the importance of considering rare pathologies during hernia surgery and underscores the role of histopathological analysis in determining the etiology of lithiasis. Reporting such cases contributes to the understanding of unusual findings, aids in surgical preparedness, and may help prevent complications. Further studies are necessary to clarify the mechanisms of stone formation in hernia sacs and to establish management protocols for similar cases.

## Data Availability

The data that support the findings of this study are available from the corresponding author upon reasonable request.
